# High resolution RNA‐seq profiling of genes encoding ribosomal proteins across different organs and developmental stages in *Arabidopsis thaliana*

**DOI:** 10.1002/pld3.320

**Published:** 2021-05-27

**Authors:** Wei Xiong, Jiancong Zhang, Ting Lan, Wenwen Kong, Xiaoyan Wang, Lin Liu, Xuemei Chen, Beixin Mo

**Affiliations:** ^1^ Guangdong Provincial Key Laboratory for Plant Epigenetics Longhua Bioindustry and Innovation Research Institute College of Life Sciences and Oceanography Shenzhen University Shenzhen China; ^2^ Key Laboratory of Optoelectronic Devices and Systems of Ministry of Education and Guangdong Province College of Optoelectronic Engineering Shenzhen University Shenzhen China; ^3^ Department of Botany and Plant Sciences Institute of Integrative Genome Biology University of California Riverside CA USA

**Keywords:** *Arabidopsis thaliana*, functional specialization, gene duplication, paralogue, ribosomal protein, ribosome heterogeneity, transcript profiling

## Abstract

In *Arabidopsis thaliana*, each ribosomal protein (RP) is encoded by a small gene family consisting of two or more highly homologous paralogues, which results in ribosome heterogeneity. It is largely unknown that how genes from multiple member containing RP families are regulated at transcriptional level to accommodate the needs of different plant organs and developmental stages. In this study, we investigated the transcript accumulation profiles of RP genes and found that the expression levels of RP genes are varied dramatically in different organs and developmental stages. Although most RP genes are found to be ubiquitously transcribed, some are obviously transcribed with spatiotemporal specificity. The hierarchical clustering trees of transcript accumulation intensity of RP genes revealed that different organs and developmental stages have different population of RP gene transcripts. By interrogating of the expression fluctuation trend of RP genes, we found that in spite of the fact that most groups of paralogous RP genes are transcribed in concerted manners, some RPs gene have contrasting expression patterns. When transcripts of paralogous RP genes from the same family are considered together, the expression level of most RP genes are well‐matched but some are obviously higher or lower, therefore we speculate that some superfluous RPs may act outside the ribosome and a portion of ribosomes may lack one or even more RP(s). Altogether, our analysis results suggested that functional divergence may exist among heterogeneous ribosomes that resulted from different combination of RP paralogues, and substoichiometry of several RP gene families may lead to another layer of heterogeneous ribosomes which also have divergent functions in plants.

## 1 INTRODUCTION

Ribosome is a ribonucleoprotein complex comprising a large and a small subunit, and is essential for catalyzing the peptidyl transferase reaction during polypeptide synthesis in all living cells. In plants, the large ribosomal subunit is composed of 48 RPL (Ribosomal Protein of Large subunit) proteins in conjunction with three rRNAs (25S, 5.8S, and 5S), whereas the small subunit is composed of 33 RPS (Ribosomal Protein of Small subunit) proteins in conjunction with the 18S rRNA (Chang et al., 2005; Savada & Bonham‐Smith, 2014). Biogenesis of cytoplasmic ribosome is a highly orchestrated process involving the coordinated production and transport of four rRNAs and 81 RPs (Saez‐Vasquez & Delseny, 2019).

In *Arabidopsis thaliana*, each RP is encoded by a small gene family containing two or more highly homologous family members (Barakat et al., 2001). The presence of multiple gene paralogues for RPs in plants, which leads to the production of heterogeneous ribosomes (Giavalisco et al., 2005), might be a consequence of high frequency of ancestral polyploidy events and could reflect a need to maintain adequate ribosome dose or to maintain some degree of ribosome heterogeneity (Blanc & Wolfe, 2004; Horiguchi et al., 2012, 2011, 2012, 2011; Martinez‐Seidel et al., 2020; Thomas et al., 2006; Xue & Barna, 2012). Recently, many studies suggested that a number of individual animal RPs have wide‐ranging extraribosomal functions in processes such as transcription, translation, mRNA processing, DNA repair, apoptosis, and tumorigenesis (Aseev & Boni, 2011; Lindstrom, 2009; Lu et al., 2015; Naora, 1999; Warner & Mcintosh, 2009; Yang et al., 2019). Although RPs were normally targeted to the nucleolus for cytoplasmic ribosomal subunit assembly with rRNAs (Degenhardt & Bonham‐Smith, 2008; Kruger et al., 2007; Lam et al., 2007; Savada & Bonham‐Smith, 2014), some RPs could be secreted out of the cell (Dai et al., 2010), all of which may suggest extraribosomal functions of RPs.

The complexity of the plant ribosome biogenesis together with extraribsomal functions of RP genes raise an important question: how the duplicated RP genes are regulated at the transcriptional level to coordinate the needs of cells for specific RPs or specific RP paralogues? By analyzing the expressed sequence tag (EST) data and the complete genomic sequence of *Arabidopsis*, previous studies have identified 249 genes (including some pseudogenes) corresponding to different cytoplasmic RP types, and found only 52 RP genes lack a matching EST accession and 19 of these contain incomplete open reading frames. These results confirm that most RP genes are expressed (Barakat et al., 2001). A group of researchers identified 996 putative RP genes spanning 79 distinct RPs in *Brassica napus* using EST data from 16 tissue collections (Whittle & Krochko, 2009). Comparative analysis of the transcript levels according to EST data for *Brassica napus* RPs revealed that a large fraction of RP genes is differentially expressed and that the number of paralogues expressed for each RP varied extensively with tissue types. Using Genevestigator (Hruz et al., 2008) to analyze *Arabidopsis* 22k microarray data, another group of researchers studied transcript levels of 192 of the 254 *Arabidopsis* RP genes and revealed that transcript levels from different RP genes show up to a 300‐fold difference across the RP population (Savada & Bonham‐Smith, 2014).

Despite these studies on the steady state levels of RP transcripts, so far, it is still not clear about how coordinated expression of RPs from multigene families is accomplished. The genome‐wide expression profiles across 79 different tissues and developmental stages using high‐throughput transcriptome sequencing (Klepikova et al., 2016) provide the opportunity to comprehensively understand the expression of the duplicated RP genes in *Arabidopsis thaliana*. In this study, we analyzed these data and found that duplicated RP genes are transcribed dynamically in different cell types with some degree of function diversity and co‐expression patterns. Furthermore, our analysis results suggested that transcript insufficiency of several RP families brings the possibility of the second layer of heterogeneous ribosomes, which may also have divergent functions.

## 2 EXPERIMENTAL PROCEDURES

### 2.1 RP gene list

The RP sequences of *Arabidopsis thaliana* were compiled based on the *Arabidopsis* Genome Initiative (AGI) identification numbers provided by previous researches (Barakat et al., 2001; Browning & Bailey‐Serres, 2015; Whittle & Krochko, 2009). We focused on a total number of 240 RP genes which have definite AGI numbers or have detected transcripts (N_RP genes_ = 256, N_Pseudogenes_ =16).

### 2.2 RNA‐seq data analysis

The RNA‐ seq data were analyzed using the pRNASeqTools. Briefly, raw reads were mapped to the Araport11 genome using STAR v2.6 (Dobin et al., 2013) with the parameters “‐‐alignIn‐ tronMax 5000 ‐‐outSAMmultNmax 1 ‐‐outFilterMultimapNmax 50 ‐‐out‐ FilterMismatchNoverLmax 0.1”. Mapped reads were counted by feature Counts v1.6.4 (Liao et al., 2014). The read counts were normalized by calculating the RPKM (Reads Per Kilobase per Million mapped reads) value using the R package edgeR (Robinson et al., 2010). RPKM=10^6^ × (reads count)/(total mapped reads) × 10^3^/(gene length; Mortazavi et al., 2008). The cDNA length of RP genes were obtained from Araport11.

### 2.3 Heatmap and hierarchical clustering

The original RPKM was log_2_ transformed. A heatmap with the hierarchical cluster tree was made using the R package pheatmap with log_2_ (RPKM) value of RP genes with the parameters “clustering_method =complete”,“clustering_distance_rows =euclidean”. （pheatmap: Pretty Heatmaps. R package version 1.0.12. https://CRAN.R‐project.org/package=pheatmap). The complete linkage method was used to cluster different samples and different RP genes. Euclidean distance was used to calculate the distance between different samples and different RP genes. Pearson correlation coefficient matrix are presented to show the correlation among 69 different organs and 10 different developmental stages.

### 2.4 Boxplot

The boxplot figure showing the overall expression level of RP genes across different organs and developmental stages was generated by the R packages ggpubr. ggpubr: 'ggplot2' Based Publication Ready Plots. R package version 0.2.5. https://CRAN.R‐project.org/package=ggpubr).

### 2.5 Calculating the transcript levels of different RP gene families

The transcript levels (RPKM value) of different RP gene families from different tissues were combined (sum of individual family members) respectively. The transcripts stoichiometry of 81 RP gene families was calculated with RPKM value of each RP gene family divided by the median RPKM value of total RP gene families of each examined tissue.

### 2.6 Expression pattern analysis

Four type of tissues (1‐day seedling(hypocotyl, cotyledons, hpical meristem with adjacent tissues), seed germination (SD.g1~SD.g3), meristem (M1~M10), flower (F1~F19.) ) which represent samples from important growth and development stages were chosen to investigate the expression pattern of RP genes. To analyse the expression pattern, RPKM of the RP genes were transformed to z‐score then clustered in six clusters by fuzzy cmeans (Futschik & Carlisle, 2005; Kumara & Futschik, 2007). R packages TCseq with the parameters“algo = cm, k = 6, standardize =TRUE” was used to obtain clustering algorithm. TCseq: Time course sequencing data analysis. R package version 1.6.1. https://bioconductor.org/packages/release/bioc/html/TCseq.html).

### 2.7 Code availability

RNA‐seq data bioinformatic analyses in this study were performed by an integrated pipeline for next‐generation sequencing analysis, pRNASeqTools v0.6 [https://github.com/grubbybio/pRNASeqTools/] in its mapping only mode to get the reads count. This pipeline can be used freely under the MIT license.

## 3 RESULTS

We downloaded the original RNA‐seq data of 79 different *Arabidopsis thaliana* tissues and developmental stages from NCBI Sequence Read Archive (SRA) (Klepikova et al., 2016). In order to maximize the representation of different organs and stages, and to provide insights into the dynamics of gene expression in the most important processes in the plant life cycle, the sequenced samples were selected from different developmental stages including seed germination, seed development, silique development, transition to flowering, flower development, ovule development. Furthermore, the samples were also chosen with special focus on tissues and stages not sampled in microarray‐based transcriptome map (Schmid et al., 2005), for example, detailed shoot apical/inflorescence meristem series and leaf development series (Klepikova et al., 2016). Each sample had two biological replicates. Description of sequenced samples is listed in Data S1. The *Arabidopsis* RP genes (Table S1) used in this research is based on previous publications (Barakat et al., 2001; Browning & Bailey‐Serres, 2015; Whittle & Krochko, 2009). Some of the RP gene AGI numbers have been updated/adjusted based on data from The *Arabidopsis* Information Resource (TAIR).

### 3.1 The expression levels of RP genes across different organs and developmental stages vary substantially in *Arabidopsis thaliana*

**FIGURE 1:**
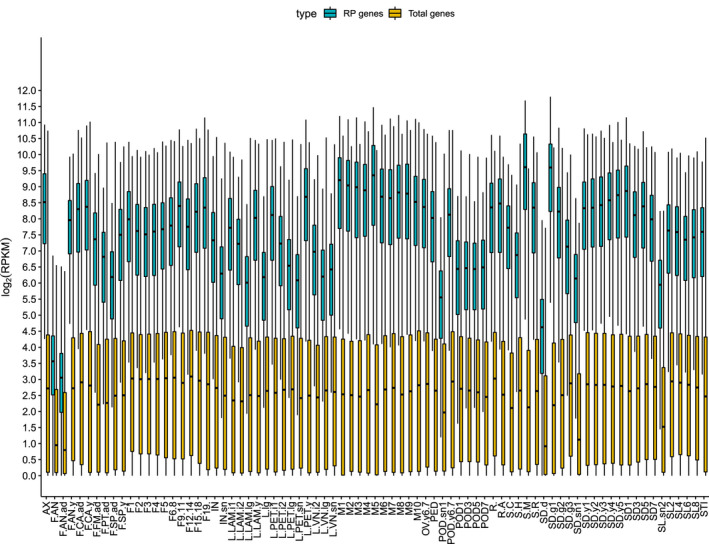
A boxplot figure showing the overall transcripts accumulation level of 240 RP genes and total genes across 79 different tissues and developmental stages in *Arabidopsis thaliana*. The average median value of RPKM (Reads Per Kilobase per Million mapped reads) of 240 RP genes and total genes were calculated from two biological replicates for each sample. The median value of log_2_(RPKM) together with upper quartile and lower quartile were plotted against different samples. Blue, RP genes; yellow, total genes

In order to understand the overall expression levels of RP genes during temporal and spatial developmental processes, we calculated the median value of RPKM (Reads Per Kilobase per Million mapped reads) of 240 RP genes and total genes across 79 different tissues or developmental stages in *Arabidopsis thaliana*. As shown in Figure 1, the median value of log_2_(RPKM) of 240 RP genes was much higher than that of total genes in all examined tissues, suggesting RP genes are transcribed at relatively high level. Comparison of the median value of log_2_(RPKM) of RP genes from different tissues (Figure 1), we found transcripts accumulation level of RP genes is varied substantially across different tissues or developmental stages. The first two lowest transcripts accumulation levels of RPs among the 79 examined tissues were observed in anthers of the mature flower (F.AN.ad) and opened anthers (F.AN), of which the median number of log_2_(RPKM) are only around 3.5. Whereas, the accumulation level of RPs from anthers of the young flower (F.AN.y), of which the median number of log_2_(RPKM) is 8, was much higher than those from the other two stages of anther development. The first two highest transcripts accumulation level of RPs among the 79 examined tissues were observed in seeds at first day after soaking (SD.g1) and meristem of 1‐day seedling (S.M), of which the median number of log_2_(RPKM) is around 10. Meristems (M), young seeds (SD.y), flowers (S), axis of the inflorescence (AX), and leaf petiole of the young leaf (L.PET.y) have relatively higher RP transcript accumulation levels than other examined tissues. In the contrast, transcript accumulation level of pod of the siliques (POD), dry seeds (SD,d), petals of the mature flower (F.PT.ad), and sepals of the mature flower (F.SP.ad) are relatively lower than those of other examined tissues. Transcript accumulation levels of RPs were correlated with the development activities of the examined tissues, suggesting that different expression level of RPs in different tissues may reflect different demands of ribosomes along with growth and development.

### 3.2 Most RP genes are ubiquitously expressed

**FIGURE 2:**
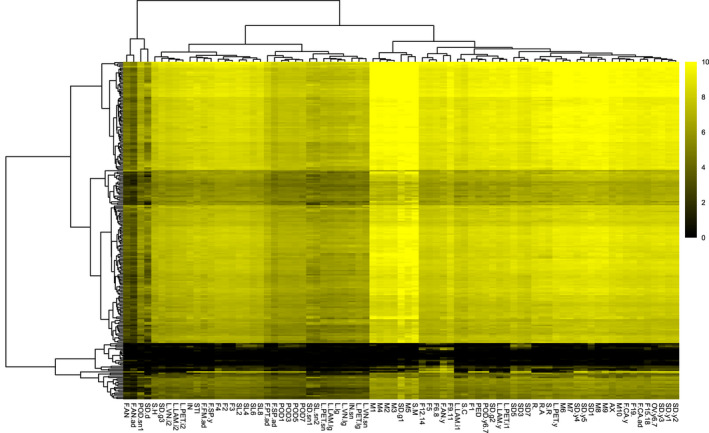
A heatmap with hierarchical cluster trees of transcripts accumulation intensity of RP genes across different organs and developmental stages. The value of log_2_(RPKM) of each RP gene was plotted against each examined sample. Yellow, relative high expression; black, relative low expression. The vertical hierarchical cluster tree shows the Euclidean distance of examined samples and the horizontal hierarchical cluster tree shows the Euclidean distance of different RP genes based on the log_2_(RPKM) value

**FIGURE 3:**
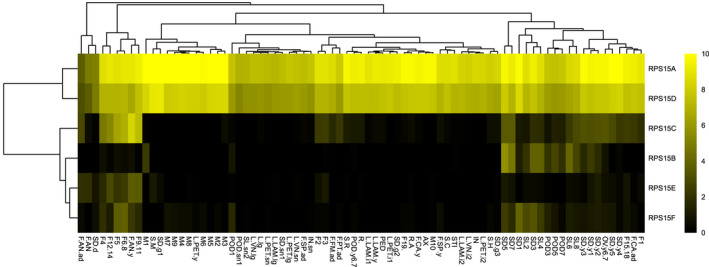
Diversified spatiotemporal transcripts accumulation pattern within *RPS15* family. A heat map with hierarchical cluster trees of transcripts accumulation intensity of *RPS15* family containing six members (*RPS15A*, *RPS15B*, *RPS15C*, *RPS15D*, *RPS15E*, and *RPS15F*) was made. Yellow, relative high expression; black, relative low expression. The vertical hierarchical cluster tree shows the Euclidean distance of examined samples whereas the horizontal hierarchical cluster tree shows the Euclidean distance of different RP genes based on the log_2_(RPKM) value

In order to investigate the transcription spatiotemporal specificity of RP genes, we made a heatmap with hierarchical cluster trees of transcript accumulation intensity of RP genes across different organs and developmental stages (Figure 2). We found that the majority of RP genes are ubiquitously expressed with exceptions that some RP genes are expressed with obviously spatiotemporal specificity. RP genes with extremely low transcripts accumulation levels or with obviously spatiotemporal specificities are shown in Figure S1. Take *RPS15* family which contains six members (*RPS15A*, *RPS15B*, *RPS15C*, *RPS15D*, *RPS15E*, and *RPS15F*) for example (Figure 3), *RPS15A*, and *RPS15D* are transcribed in all examined tissues, whereas the other four members are specifically transcribed in certain reproductive organs: *RPS15B* are only transcribed in developing seeds ((SD3), (SD5), (SD7)) and developing siliques ((SL4), (SL6)), *RPS15C* are only transcribed in anthers of the young flower (F.AN.y), several stages of flower development ((F4), (F5), (F6‐8), (F9‐11), (F12‐14)) and two stages of developing seeds ((SD5), (SD7)), *RPS15E* are only transcribed in anthers of the young flower (F.AN.y), only one stage of flower development (F9‐11), *RPS15F* are only transcribed in two stages of flower development ((F5), (F6‐8)) and one stage of developing seeds (SD1). Divergence in spatiotemporal specificity of paralogous RP genes transcription suggests they could have undergone functional specializations among them. Spatiotemporally specific expression of paralogous RP genes lead to the formation of spatiotemporally specific ribosomes which may be needed for certain mRNA translation, thus could play a role in specific organ establishment and/or tissue‐specific physiology.

The hierarchical clustering tree of transcripts accumulation intensity of 240 different RP genes revealed that distinct members from different RP families cluster together, implying that these non‐paralogous RPs have similar expression intensity crossing different organs and developmental stages. Meanwhile, the hierarchical clustering tree of the examined tissues reflected an organ‐specific and developmental stage‐specific structure, as the different tissues series are found to be organized into distinct clades. For example, different parts of root, different parts of leaf, different stages of meistems are clustered together, respectively. Thus, different tissues and developmental stages have distinct population of RP gene transcripts. Ribosomes are highly heterogenous in *Arabidopsis,* each organ or developmental stage may need different association of non‐paralogous RPs.

### 3.3 Stoichiometric analysis of steady‐state level of mRNAs between different RP families

Any given ribosome contains only a single polypeptide of most RPs except acidic ribosomal proteins P1 and P2 which form a heterodimer and two heterodimers are present per 60S subunit (Armache et al., 2010; Inglis et al., 2019). However, each one of the 81 *Arabidopsis* RPs is encoded by two or more paralogues. In order to maintain the appropriate stoichiometry of ribosome components, the amount of each RP family must be regulated at comparable level. Therefore, the transcript levels of RP gene families may be coordinately controlled. In this study, we combined transcript levels of each RP gene family (sum of individual family members) of each different tissue. The stoichiometry of 81‐RP‐family transcripts was calculated with RPKM value of each RP gene divided by the median RPKM value of total RP genes of each examined tissue. As shown in Data S2, the values of most RP gene families are around 1 (>0.5 meanwhile <2) but the value of several RP genes are largely deviated from 1 (<0.5 or >2), indicating that the expression level of most RP gene families are well‐matched but some are obviously higher or lower. For instance, the value of the *RPL9* gene families is less than 0.5 in almost all examined tissues (Data S2); the value of the gene family *RPL10* is greater than 2 in opened anthers (F.AN), pods of the senescent silique (POD.sn1), dry Seeds (SD.d), senescent siliques (SL.sn2), (*t*‐test, *p* < .05); the value of the gene family *RPL14* is greater than 2 in senescent internodes (IN.sn), germinating seeds (SD.g2), veins of the mature leaves (L.VN.lg), carpels of 6th and 7th flowers (POD.y6‐7), petioles of the mature leaves (L.PET.lg), petiole of the senescent leaves (L.PET.sn), pedicels (PED), developing flowers ((F3), (F4), (F5), (F6.8), (F12.14), (F19.), (*t*‐test, *p* < .05)); the value of the gene family *RPL19* is greater than 2 in pods of the senescent siliques (POD.sn1), senescent internodes (IN.sn), anthers of the mature flowers (F.AN.ad), opened anthers (F.AN), petiole of the senescent leaves (L.PET.sn), mature leaves (L.lg), veins of the mature leaves (L.VN.lg), lamina of the mature leaves (L.LAM.lg), (*t*‐test, *p* < .05); the value of the gene family RPS17 is greater than 2 in anthers of the mature flowers (F.AN.ad) but is significantly less than 0.5 in seedling meristems (S.M), developing seeds (SD.y1, SD.y2, SD.y5), senescent internodes (IN.sn), (*t*‐test, *p* < .05), (Data S2, and Figure S2). As for the acidic ribosomal proteins, the value of the gene family *RPLP1* is greater than 2 in the mature flowers (F.AN.ad), seedlings meristems (S.M), meristems at 10 days after germination (M5), opened anthers (F.AN) and dry seeds (SD.d), and the value is greater than 1.5 in most examined samples (*t*‐test, *p* < .05), (Data S2, and Figure S2). The value of the gene family *RPLP2* is greater than 1 in all samples and is greater than 1.5 in near half of the examined tissues (*t*‐test, *p* < .05), (Data S2, and Figure S2). The value of the plant specific gene family *RPLP3* is >0.5 meanwhile <1 in all of the examined tissues (*t*‐test, *p* < .05), (Data S2).

**FIGURE 4:**
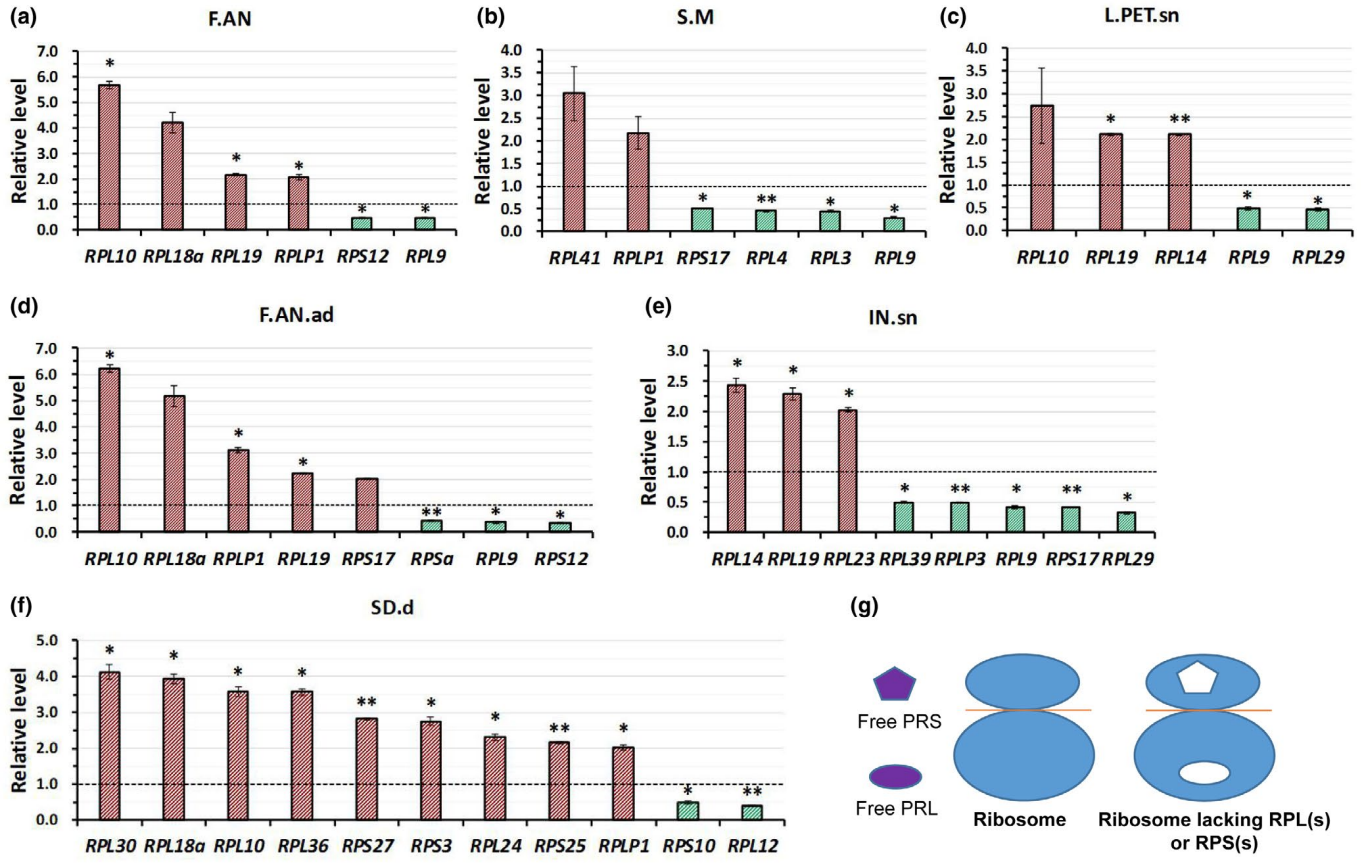
Samples of which the number of RP gene families largely deviating from 1 (<−0.5 or >2) is more than 5. Examined tissues such as (a) F.AN; (b) S.M; (c) L.PET.sn; (d) F.AN.ad; (e) IN.sn; (f) SD.d, of which the number of RP gene families largely deviating from 1 (<−0.5 or >2) is more than 5. (g), proposed hypothesis of free RPs which may act outside ribosomes and heterogeneous ribosomes contributed by some substoichiometric RPs. Relative level was calculated with RPKM value of each RP gene divided by the median RPKM value of total RP genes in each examined tissue. Error bars indicate SD from two independent experiments; asterisk indicates significant difference (*t*‐test, *p* < .05)

Interestingly, we found numbers of RP gene families, of which the values are greatly deviated from 1 (<0.5 or >2), are much larger in dry seeds (SD.d), senescent internodes (IN.sn), anthers of the mature flowers (F.AN.ad), and opened anthers (F.AN), where destructive metabolisms are the major biochemical reactions, than other examined tissues (Figure 4), suggesting that the stoichiometries of RP genes transcript accumulation levels are highly unequal in these tissues. The translation efficiencies of RP gene transcripts maybe different from each other (Fernie & Stitt, 2012). On what degree the stoichiometry inequality of RP gene transcripts affect RP stoichiometry at protein level needs to be investigated. RP families with higher transcript accumulation levels may have more RP proteins being translated, thus the amount of them maybe more than enough for the assembly of ribosome (the proposed hypothesis is shown in Figure 4g), suggesting these free RPs may act outside ribosomes. In contrast, RP families with extremely lower transcripts accumulation levels may have less RP proteins being translated, therefore they maybe not sufficient to be incorporated into ribosomes, causing a portion of ribosomes lacking these RPs (the proposed hypothesis is shown in Figure 4g). Apart from the heterogeneity of ribosomes contributed by different combination of non‐paralogous RPs, substoichiometry of RP genes causes another layer of ribosome heterogeneity. Ribosomes lacking one or more RP(s) may not recognize some kinds of mRNAs or may have lower translating efficiency toward specific subpools of mRNAs as reported in mammalian cells (Kondrashov et al., 2011; Shi et al., 2017). Together, heterogeneous ribosomes contributed by some substoichiometric RPs and free RPs resulted from several superfluous RPs that are not incorporated into ribosomes, may play roles in regulating specific developmental stages or participating the establishment of specific organs.

### 3.4 Synchronous and discrepant expression patterns coexist for RP genes

**FIGURE 5:**
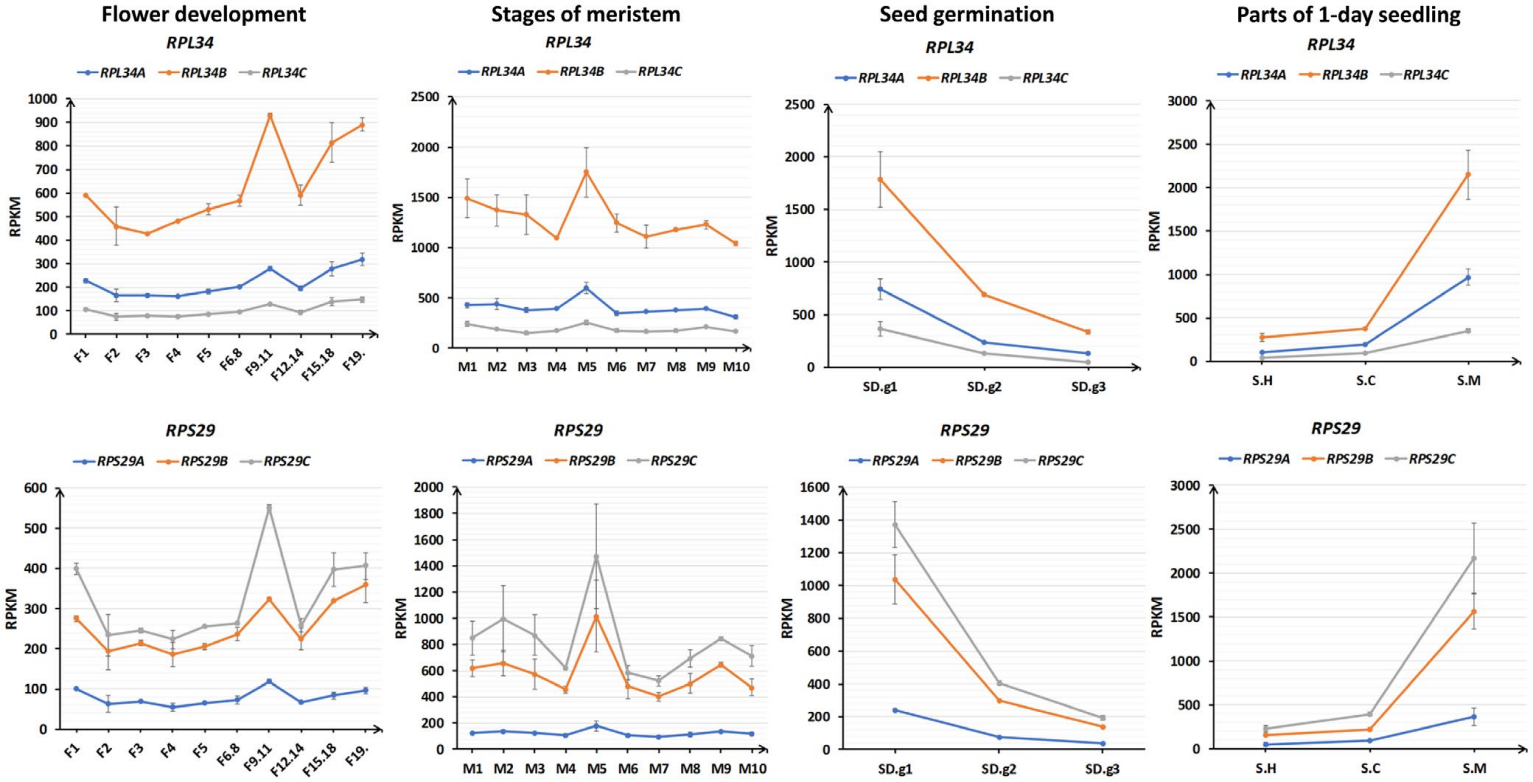
Concerted expression patterns of paralogous RP genes. The expression fluctuation trend of RP genes was investigated from four types of tissues representing important growth and development stages (Parts of 1‐day seedling (hypocotyl, cotyledons, apical meristem with adjacent tissues); seed germination (SD.g1~SD.g3); meristem (M1~M10), and flower development (F1~19.)). *RPL34* and *RPS29* families were used as examples of RP gene paralogues with concerted expression pattern

**FIGURE 6:**
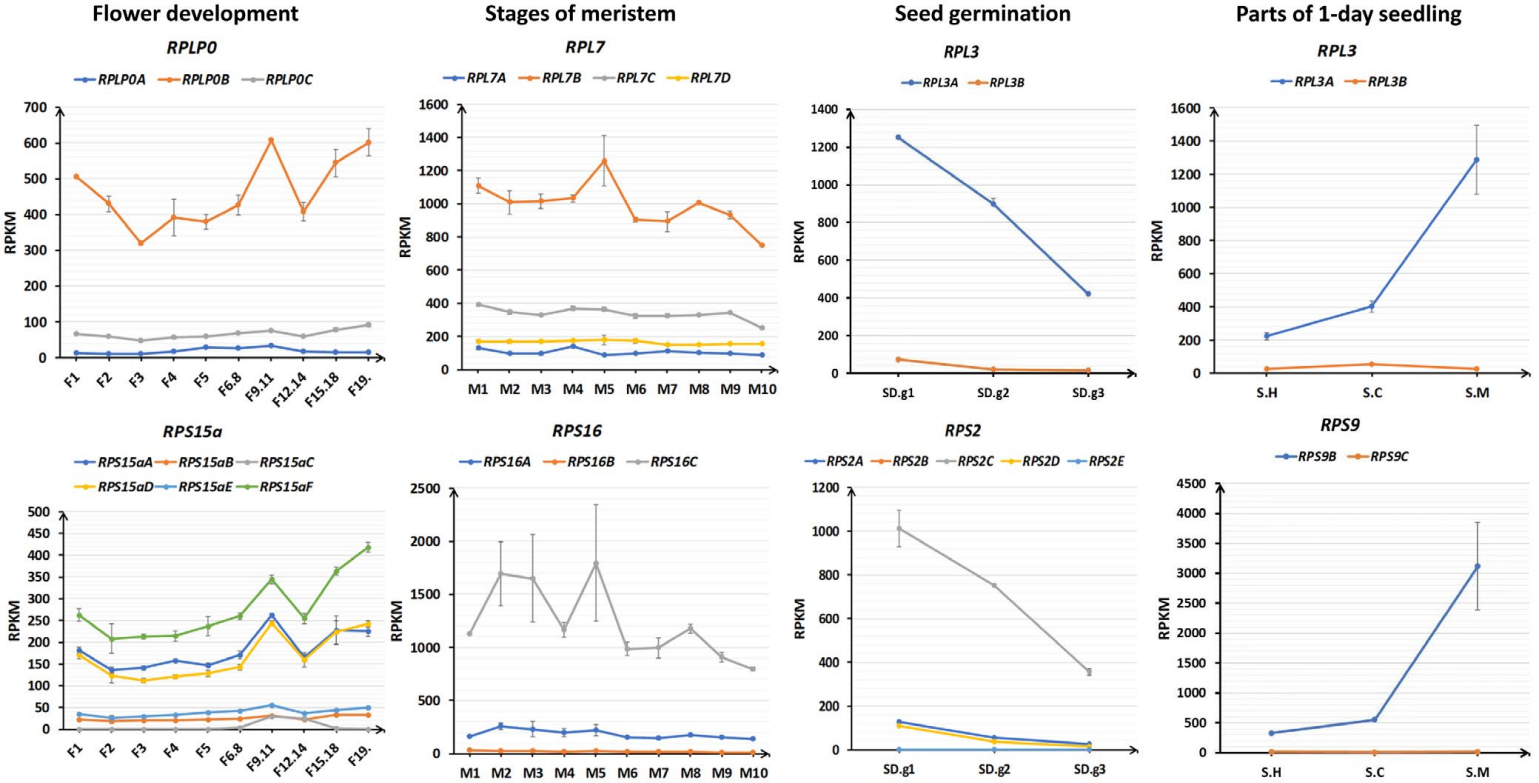
Contrasting expression patterns of paralogous RP genes. Families such as *RPLP0*, *RPL3*, *RPL7*, *RPS2*, *RPS9*, *RPS15a,* and *RPS16* were used as examples of RP gene paralogues with contrasting expression pattern

Former works investigating expression patterns of paralogous RP genes only compared the expression levels among them, RP families in *Arabidopsis* have been classified into two groups based on the transcript levels of their individual members (Type I, RP families with members having similar levels of transcript accumulation; Type II, RP families with members having varied levels of transcript accumulation; Savada & Bonham‐Smith, 2014). In this study, in order to further understand the expression patterns of paralogous PR genes, we drew line charts comparing RPKM values of RP genes over different tissues or the same tissue over different developmental stages. Four types of tissues at the same developmental stage (parts of 1‐day seedling, hypocotyl, cotyledons, and apical meristem with adjacent tissues); same tissues at different developmental stage (germinating seeds (SD.g1‐SD.g3)); shoot meristems (M1‐M10); flowers (F1‐F19.)), which represent samples from important growth and developmental stages, were chosen. For the majority of RP families (Table 1), their paralogous PR genes are transcribed in concerted manners. Take *RPL34* and *RPS29* families for example, although the expression levels of these paralogous PR genes are different, the expression fluctuation trends of them are well matched across different tissues and developmental stages (Figure 5), suggesting they are regulated in concerted manners, thus may demonstrate that these paralogous RP genes are functional redundant to maintain adequate dosages of their families. Meanwhile, for some RP families (Table 1), their members are transcribed in different manners. For example: *RPLP0*, *RPL3*, *RPL7*, *RPL15*, *RPS2*, *RPS9*, *RPS15a*, and *RPS16* are found to have discrepant expression fluctuation trends (Figure 6), suggesting they are transcribed in distinctive manners with the possibility of diversified functions. Compensatory drift model envisions that although dosage‐balance selection constrains total expression of paralogous genes to the optimum level, expression of each paralogue can diverge by drifting from its original level (Thompson et al., 2016). Our analysis results suggested that a portion of paralogous RP genes might have undergone functional specialization whereas the majority of paralogous RP genes seemed to remain the same function.

**TABLE 1:** Expression patterns within RP families

Similar pattern		Different pattern
*RPL4*	*RPS6*	*RPL3*
*RPL5*	*RPS7*	*RPL7*
*RPL6*	*RPS10*	*RPL8*
*RPL7a*	*RPS11*	*RPL10*
*RPL9*	*RPS12*	*RPL11*
*RPL10a*	*RPS13*	*RPL13a*
*RPL12*	*RPS14*	*RPL14*
*RPL13*	*RPS17*	*RPL17*
*RPL15*	*RPS18*	*RPL18*
*RPL18a*	*RPS20*	*RPL19*
*RPL22*	*RPS21*	*RPL21*
*RPL23*	*RPS24*	*RPL23*
*RPL23a*	*RPS26*	*RPL24*
*RPL26*	*RPS27*	*RPL27*
*RPL28*	*RPS28*	*RPL27a*
*RPL29*	*RPS29*	*RPLP0*
*RPL34*	*RPS30*	*RPLP1*
*RPL35a*		*RPLP2*
*RPL36*		*RPL30*
*RPL36a*		*RPL31*
*RPL37*		*RPL32*
*RPL37a*		*RPL35*
*RPL38*		*RPS2*
*RPL39*		*RPS8*
*RPL40*		*RPS9*
*RPL41*		*RPS15*
*RPLP3*		*RPS15a*
*RPSa*		*RPS16*
*RPS3*		*RPS19*
*RPS3a*		*RPS23*
*RPS4*		*RPS25*
*RPS5*		*RPS27a*

Note

RP family, with all members are transcribed in concerted manner, is listed in the “Similar pattern” group; whereas RP family, with one or more member(s) has/have different transcription manner(s), is listed in the “Different pattern” group.

**FIGURE 7:**
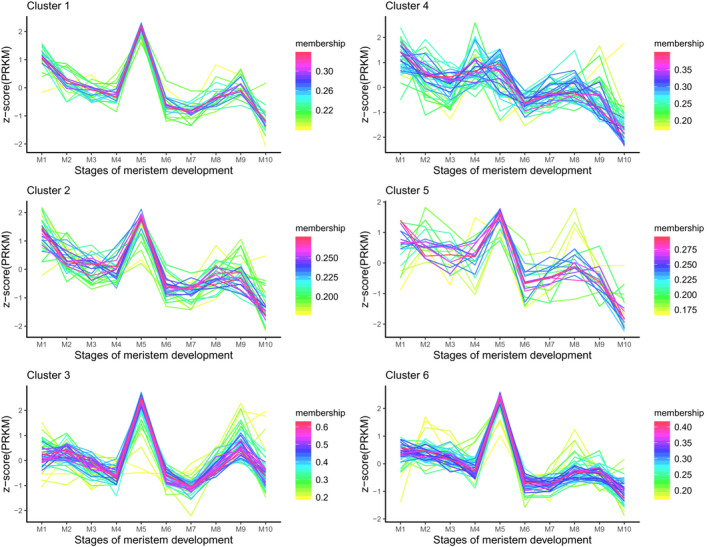
Consensus expression patterns of non‐paralogous RP genes in different developmental stages of meristem. The RPKM value of paralogous and non‐paralogous RP genes from different developmental stages of meristem was treated in accordance with Z‐score using R packages TCseq with the parameters“algo = "cm", k = 6”s

We also compared the expression fluctuation trend between different RP gene families. Interestingly, we found that the expression fluctuation trends of most RP genes from different families are similar (Figure 7, Figures S3–S5), indicating they are regulated in highly coordinated manners. Thus, synchronous expression of RP genes may play an important role in ribosome biogenesis. It was reported that almost half of all *Arabidopsis* RP genes carry several clustered GCCCR motifs in their proximal promoters, which could be recognized by a transcription factor called TCP20 (Li et al., 2005). TCP20 mediated transcriptional regulation of RP gene expression is likely to contribute to the synchronous expression of RP genes.

## 4 DISCUSSION

### 4.1 The complexity of RP gene expression patterns in *Arabidopsis*

mRNAs are translated into proteins by ribosomes which are composed of rRNAs and RPs in all living creatures. Genes encoding RPs are generally regarded as the housekeeping components of the cell to accommodate translational needs in development stages or growth situations, and are widely used for the standardization of transcript analysis data (Nicot et al., 2005; Sterky et al., 2004). In *E. coli*, RP genes are clustered together and are arranged into 20 operons with approximately half of the genes mapping to a single locus (Mager, 1988). The arrangement of prokaryotic RP genes in operons provides a regulatory strategy which ensures coordinated expression and simultaneous regulation of groups of RP genes. Under normal growth situations, coordinated expression of prokaryotic RPs ensures ribosome biogenesis proportional to the growth rate, without significant accumulation of unincorporated ribosomal constituents. The expression of prokaryotic RP genes could be rapidly changed in response to stimuli such as nutrient availability (Nomura, 1999; Schmid et al., 2005). In yeast *Saccharomyces cerevisiae*, three‐fourths (59/79) of the RPs are encoded by functionally duplicated genes of which are not transcribed at the same level Jimenez et al., 2003; Warner et al., 1985). In mammals, most functional RPs are encoded by a single gene although there are about 2,000 pseudogenes that maybe related to RP genes (Balasubramanian et al., 2009). It should be noted that few mammalian RP genes are encoded by more than one functional paralogues. For example, in human there are three paralogous genes encoding *RPS4,* namely *RPS4X*, *RPS4Y1,* and *RPS4Y2,* which are located on the X chromosome and the Y chromosome respectively (Ellis et al., 2010; Fisher et al., 1990). *RPS4X* and *RPS4Y1* are found to be ubiquitously expressed; in contrast, the expression of *RPS4Y2* is restricted to the testis and prostate, suggesting functional specialization of these paralogues.

The existence of multigenes for each RP family in plants presents a picture of RP expression that may be more complex than those that were previously described for other species. The expression patterns of some RP genes have been investigated, primarily at the level of transcript abundance (Dresselhaus et al., 1999; Hulm et al., 2005; McIntosh & Bonham‐Smith, 2005). However, the overall profiles of the coordinate response of RP genes to cell differentiation, growth, development remains to be comprehensively investigated. Achievement of such objective requires the development of new genetic and genomic resources. The genome‐wide expression profiles across different tissues and developmental stages obtained from high‐throughput transcriptome sequencing allow us to visualize and accurately understand RP gene expression across different organs and developmental stages.

### 4.2 Different organs and developmental stages demand different amount of ribosomes

Our analysis results revealed that transcripts accumulation levels of RP genes vary substantially across different organs and developmental stages. Anthers of mature flower (F.AN.ad) and opened anthers (F.AN), which are considered as highly differentiated organs, were found to have the lowest transcripts accumulation level of RP genes among the 79 examined samples with the median number of log_2_(RPKM) being only around 3.5. Whereas, anthers of the young flower (F.AN.y), of which the median number of log_2_(RPKM) is 8, have much higher transcripts accumulation levels of RP genes than those two development stages. RP transcripts accumulation level from dry seeds (SD.d) at dormant state, was the 3rd lowest among the examined samples. Thus, it is reasonable that the expression level of RPs from established and quiescent tissues are relatively low, a reduced level of ribosome is still sufficient to support the living of them. After soaking, seeds begin to germinate, the expression level of RPs from seeds at first day after soaking (SD.g1) was the first highest among the 79 examined samples cross different organs and different developmental stages. During the early stage of seed germination, initial protein synthesis is dependent on residual ribosomes within the cells of mature dry embryos, but newly translated RPs are incorporated into ribosomes within hours of initial protein synthesis (Dommes & Walle, 1990). This developmental stage must require substantial amount of newly synthesized proteins such as transcription factors, enzymes, et al., thus must demand extensive ribosomes, which well explains the highest expression levels of RP genes.

### 4.3 Varied combination of RP paralogues in different cell types may regulate the selection of mRNA translation subpools

The heat map with hierarchical clustering trees of RP genes across the 79 different tissues and different developmental stages based on the intensity of high‐throughput transcriptome sequencing suggests that RP gene transcript population changes dramatically with tissue types. A number of RP genes are ubiquitously transcribed, whereas some RP genes are only transcribed in specific tissues. Tissue specific RPs could be divided into two groups: one group of RPs are only transcribed in limited tissues; another group of RPs are transcribed in most examined tissues but not transcribed in few tissues. We speculate that ubiquitously expressed RPs may assemble into ribosomes which satisfy the basic protein translation demands for cell survival. Tissue specific RPs may play a regulatory role in gene/protein expression through heterogeneous ribosomes lacking or containing different RP paralogues. The participation of heterogeneous ribosomes in gene regulation has been reported recently among yeast strains (Komili et al., 2007). Furthermore, highly diverse and distinctive RP paralogue combinations observed in many examined tissues indicates that duplicated RP genes may have developed into functionally distinct paralogues associated with distinct tissues, and differentially expressed during plant development. This observation is consistent with the ribosome filter hypothesis, which postulates that rRNAs, and the combination of different RP paralogues, regulate the selection of translating supools of transcripts in different cell types, participating in cell differentiation and organ establishment in an elaborate way (Mauro & Edelman, 2002).

### 4.4 Potential ribosome heterogeneity resulted from substoichiometry of RPs

The appropriate stoichiometry of ribosomal proteins in the nucleolus is very important for efficient ribosome biogenesis. It is generally considered that equal molar ratio of each RP family exists in assembled ribsomes. Our analysis results reveal that transcript accumulation levels of most RP gene families are well‐matched but some are obviously higher or lower. What needs to be investigated is whether RPs with lower accumulated transcripts are limiting factors in ribosome biogenesis or whether there is a mechanism to compensate the lower transcript level by higher translational efficiency. However, it is notable that recently a group of researchers applied a quantitative mass spectrometry approach to measure the absolute abundance of subsets of core RPs and identified four RPs (RPL10A, RPL38/eL38, RPS7/eS7, and RPS25/eS25) with an 30%–40% depletion within mouse embryonic stem cells (Shi et al., 2017), which are significantly substoichiometric in polysomes. In addition to the plant ribosome heterogeneity caused by different combination of non‐paralogous RPs, substoichiometry of RP genes bring another layer of ribosome heterogeneity. Heterogeneity results from substoichiometry of RPs endows ribosomes with different selectivity for translating subpools of transcripts, including those controlling metabolism, the cell cycle, and development in animals (Shi et al., 2017). We suspect that substoichiometry of some RPs may also exist in *Arabidopsis* ribosomes according to our findings that transcripts accumulation levels of some RPs are obviously lower. Alternatively, RP families with higher transcripts may have more protein products and may be required for extraribosomal functions in addition to their conventional role in ribosome biogenesis and function. For example, it was reported that *RPL24* play an important role in miRNA biogenesis in *Arabidopsis* (Li et al., 2017). According to our analysis result, the expression level of *RPL24* family was a little bit higher than the median level of total RP families in most examined tissues (Data S2), which could be the evidence supporting the existence and action of free RPL24 outside the ribosome. RPL10, which was demonstrated to act in antivirus defense (Zorzatto et al., 2015), was another example of extraribosomal function of RPs. Our analysis results showed that expression level of RPL10 family are obviously higher than expected from a stoichiometric view of ribosome organization (Figure S2 and Data S2).

### 4.5 Functional redundant and functional specialization co‐exist within paralogous RPs

Duplication of RP genes is originated from gene or genome duplication events (Blanc & Wolfe, 2004). Gene balance pressures together with dosage effects could explain the high rates of retention of duplicated RP genes in the genome (Birchler et al., 2001, 2007, 2001, 2007; Conant & Wolfe, 2008; Veitia et al., 2008). The non‐allelic non‐complementation phenomenon, which may indicate dosage effects between paralogous genes, was found in members within RP families, such as *RPL5* (Fujikura et al., 2009), *RPS6* (Creff et al., 2010), *RPL4* (Rosado et al., 2010), *RPL36a* (Casanova‐Saez et al., 2014), and *RPL23a* (Xiong et al., 2020). The transcriptional fluctuation trends of some paralogous RP genes across different tissues and developmental stages was found well‐matched although the transcript accumulation levels between them maybe different, suggesting some paralogous RP genes are regulated in the concerted manner. Studies demonstrated that the loss‐of‐function *rpl36ab* and *rpl36aa* mutations have similar phenotypes and combined effects of haploinsufficiency and purifying selection drive retention of these two paralogs in *Arabidopsis* (Casanova‐Saez et al., 2014). Our analysis results showed that paralogous *RPL36a* genes are transcribed in a concerted manner. Knockdown of *RPL23aA* resulted in severely developmental phenotypes whereas knockout of *RPL23aB* has no obvious phenotype (Degenhardt & Bonhamsmith, 2008a, 2008b, 2008a, 2008b). Interestingly, *RPL23aA* and *RPL23aB* were found to be transcribed in a concerted manner with much higher expression level of *RPL23aA* than that of *RPL23aB,* and over‐expression of *RPL23aB* in *rpl23aa* could rescue the phenotype of *rpl23aa* (Xiong et al., 2020). We thought paralogous RP genes with concerted expression patterns may be the evidence supporting the dosage hypothesis which posits that the presence of duplicated RP genes might be necessary to maintain adequate RP dosages with functional redundant. Meanwhile, we also found a lot of paralogous RP genes are transcribed in highly contrasting manners, indicating function divergences exist within these paralogous RP genes. Plant paralogous *RPL10* genes were found to have different roles during development and responds differently to ultraviolet‐B stress (Ferreyra, Biarc, et al., 2010; Ferreyra, Pezza, et al., 2010). Consistent with genetic and functional studies of paralogous *RPL10* genes, our analysis results revealed that paralogous *RPL10* genes have contrary expression patterns (Table 1). Divergence in expression patterns is believed to be an important evidence proving functional specialization of duplicated genes (Adams et al., 2003; Blanc & Wolfe, 2004; Casneuf et al., 2006). Together, our analysis results suggested that functional redundant and functional specialization may co‐exist within paralogous RPs.

## ACCESSION NUMBERS

The original RNA‐seq data of *A. thaliana* different organs and developmental stages were downloaded from NCBI Sequence Read Archive (project ID PRJNA314076 for samples except meristem and project ID PRJNA268115 for the meristem samples).

## CONFLICT OF INTEREST

The authors have declared no conflict of interest.

## AUTHOR CONTRIBUTIONS

BM, XC, and WX designed this work; JZ, TL, WK, and XW analyzed the RNA‐seq data; WX and LL analyzed the results; WX, JZ, BM, and XC wrote the manuscript. All authors agreed to be accountable for the content of the work.

## Supporting information

Fig S1

Fig S2

Fig S3

Fig S4

Fig S5

Table S1

Data S1

Data S2

Data S3

Data S4
