# Supplementary figures and images for: High resolution RNA‐seq profiling of genes encoding ribosomal proteins across different organs and developmental stages in Arabidopsis thaliana
Source: Plant Direct. 2021 May 27;5(5):e00320. doi: 10.1002/pld3.320 (PMC8156134; doi:10.1002/pld3.320)

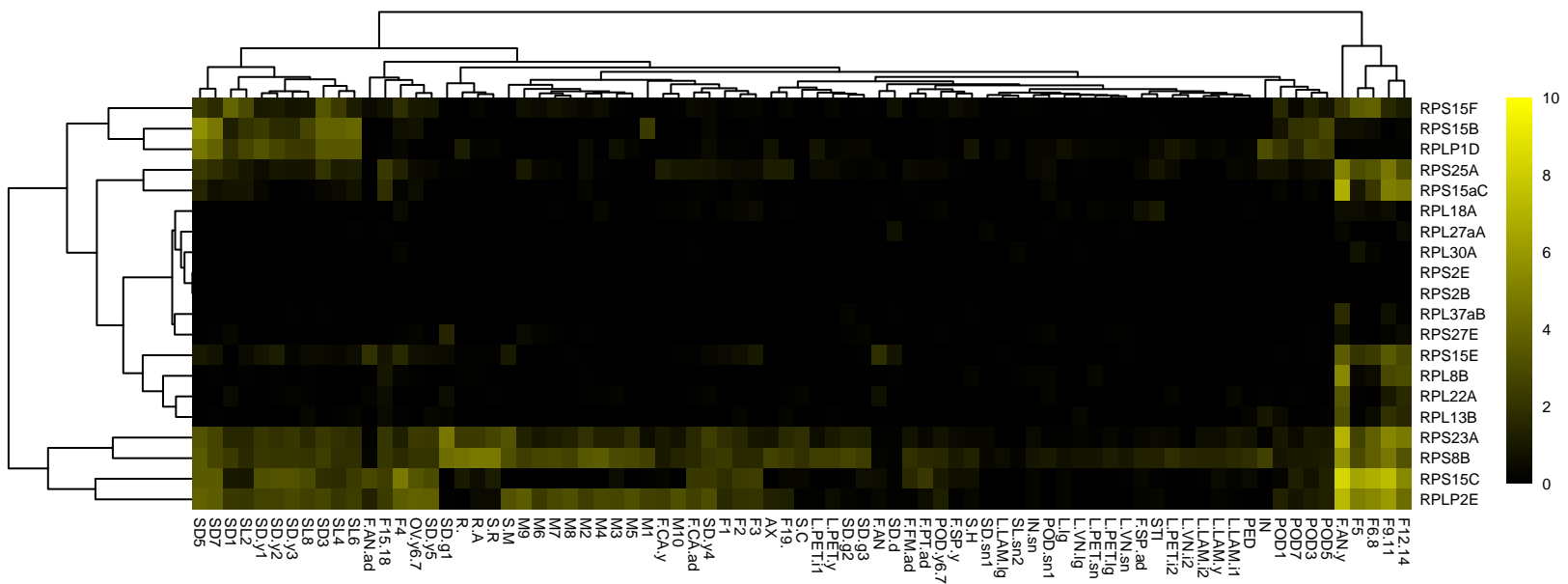

Supplement: Supplementary file 1 — Fig S1 [file PLD3-5-e00320-s009.pdf]

**(a)***RPL19*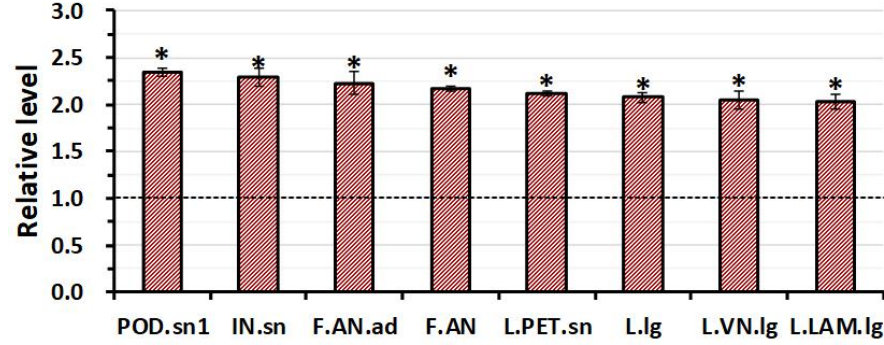**(b)***RPS17*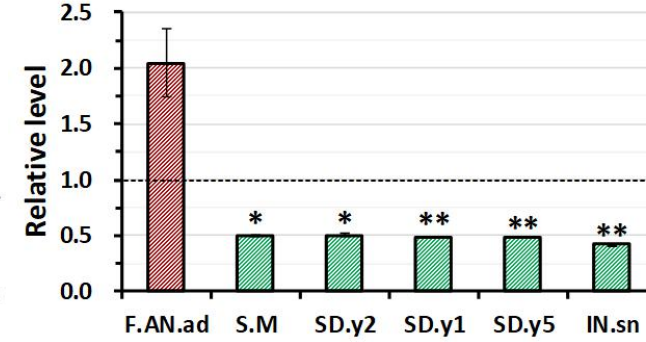**(c)***RPL10*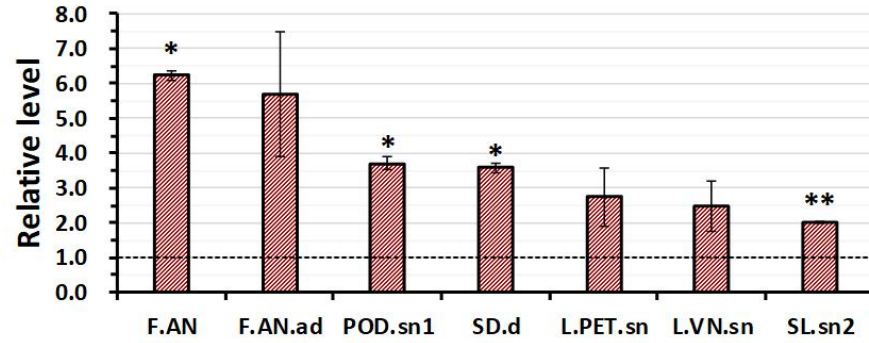**(d)***RPLP1*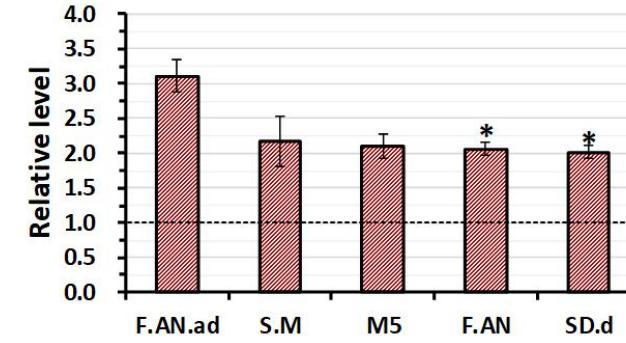**(e)***RPL14*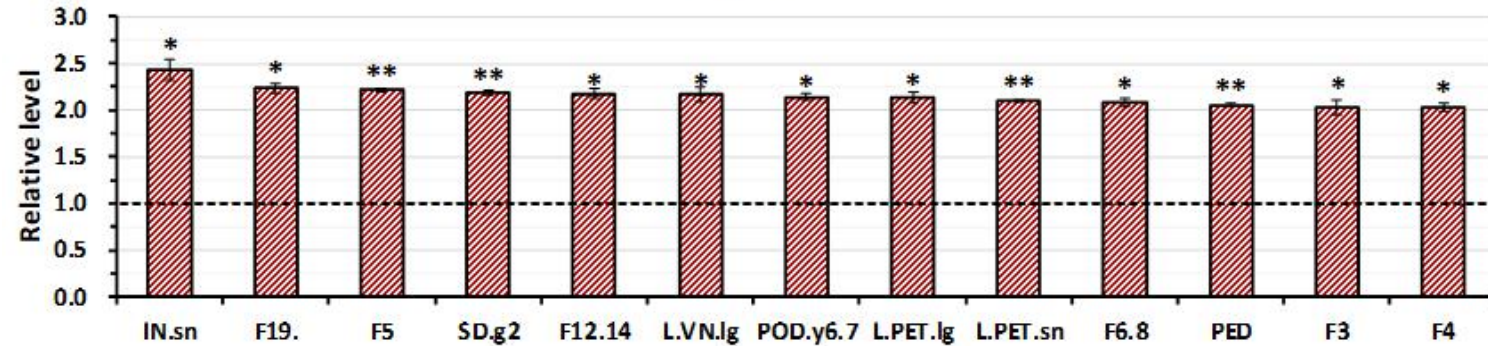

Supplement: Supplementary file 2 — Fig S2 [file PLD3-5-e00320-s005.pdf]

Cluster 1

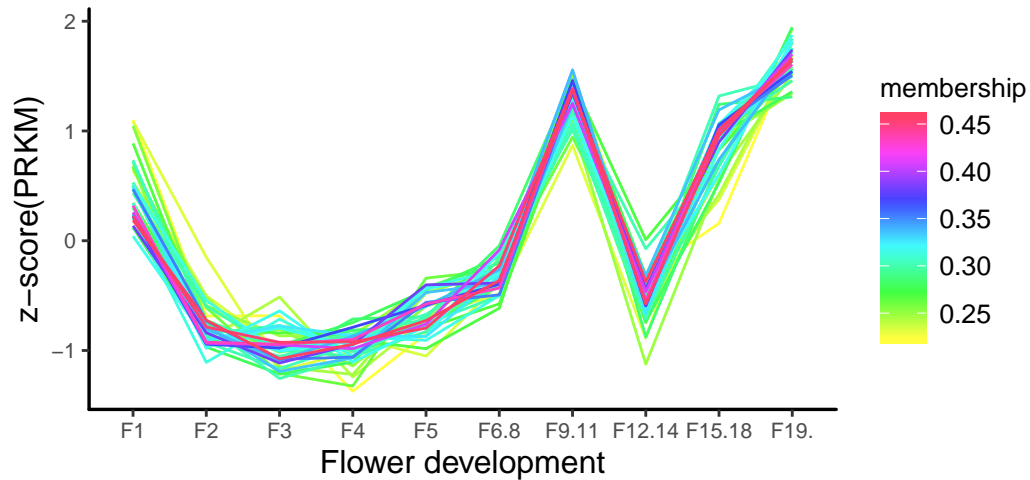

Cluster 4

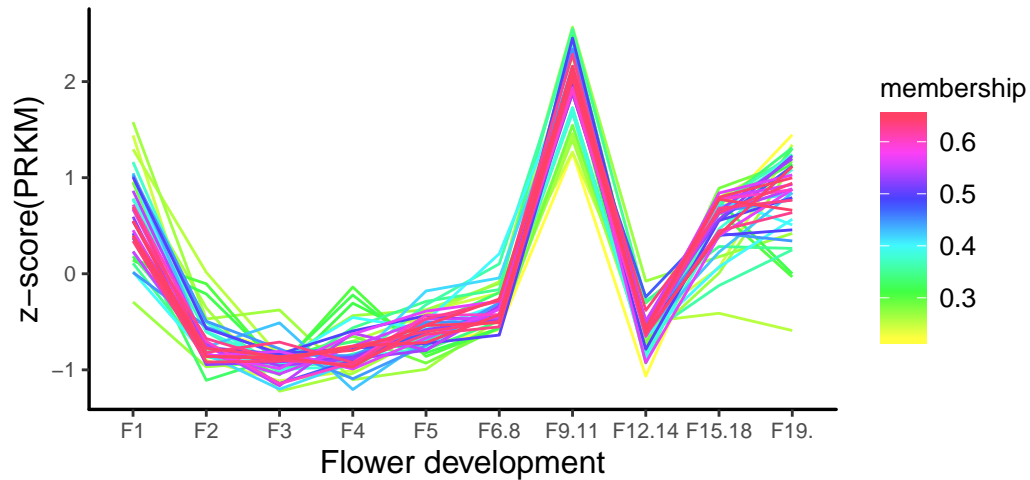

Cluster 2

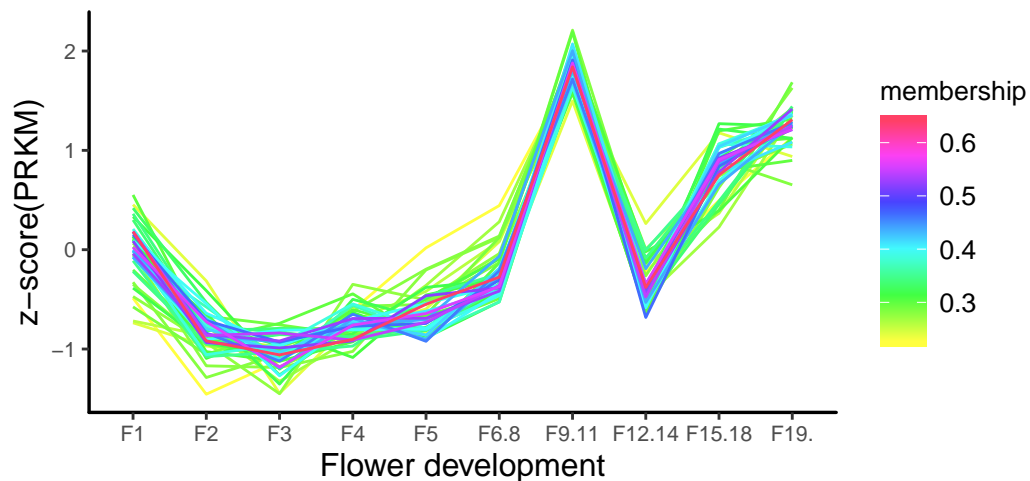

Cluster 5

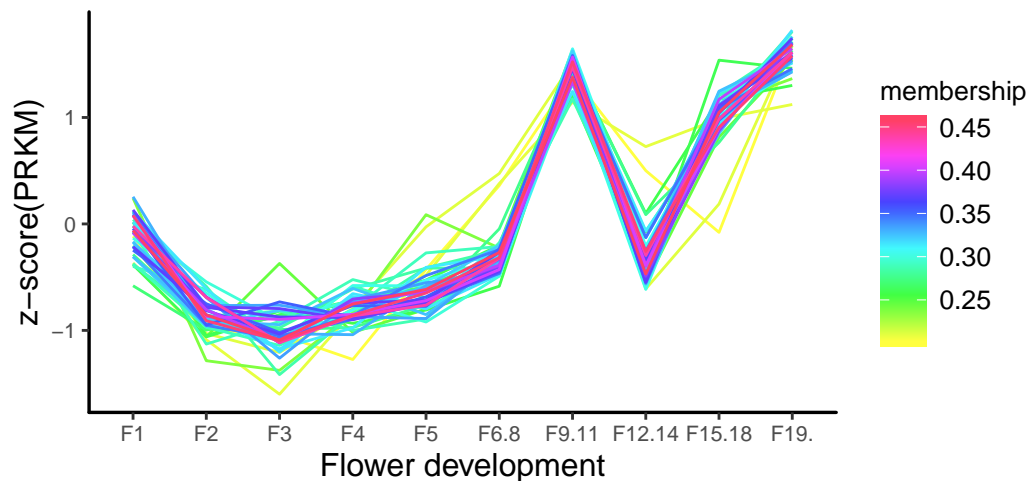

Cluster 3

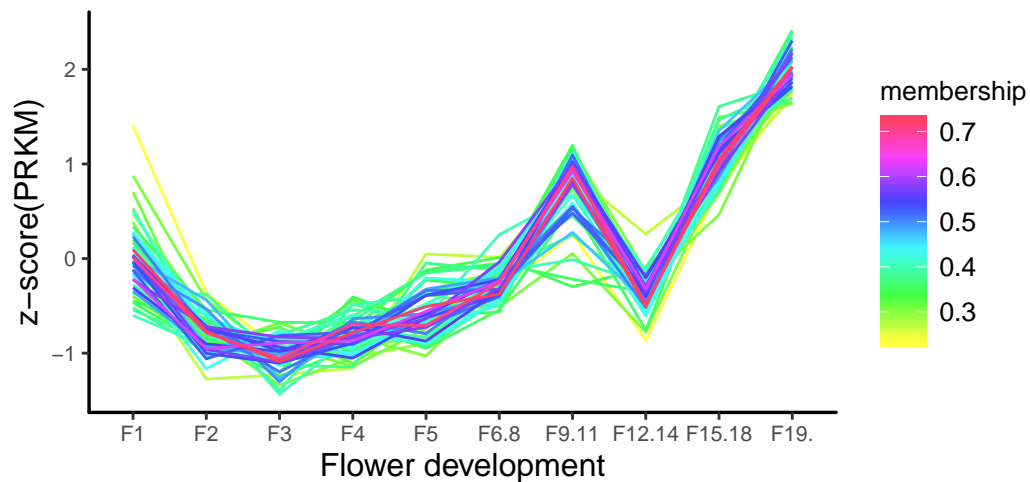

Cluster 6

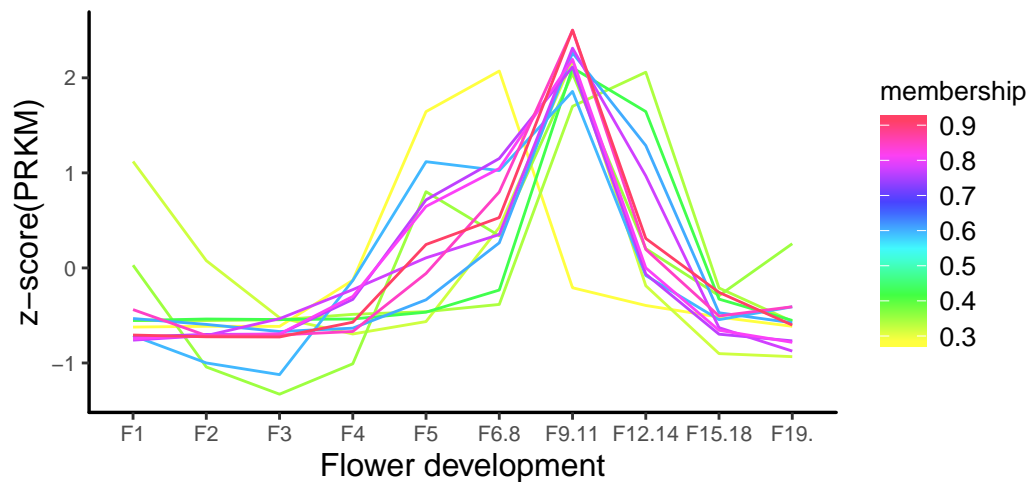

Supplement: Supplementary file 3 — Fig S3 [file PLD3-5-e00320-s001.pdf]

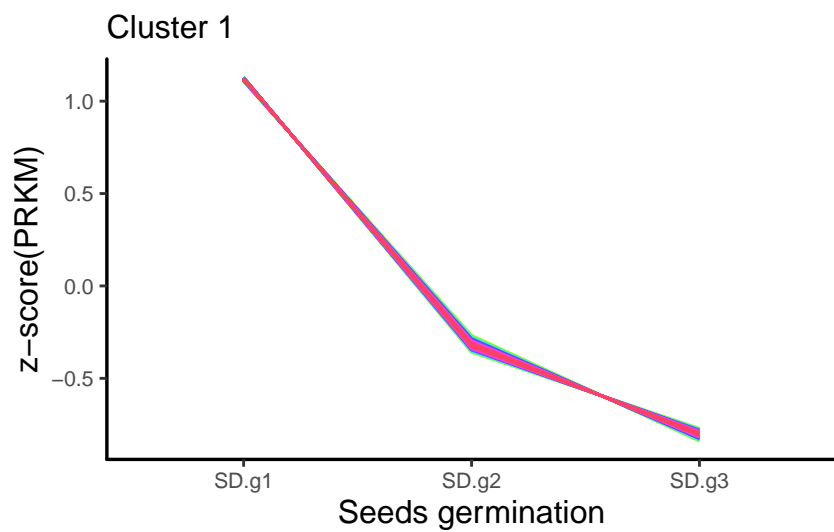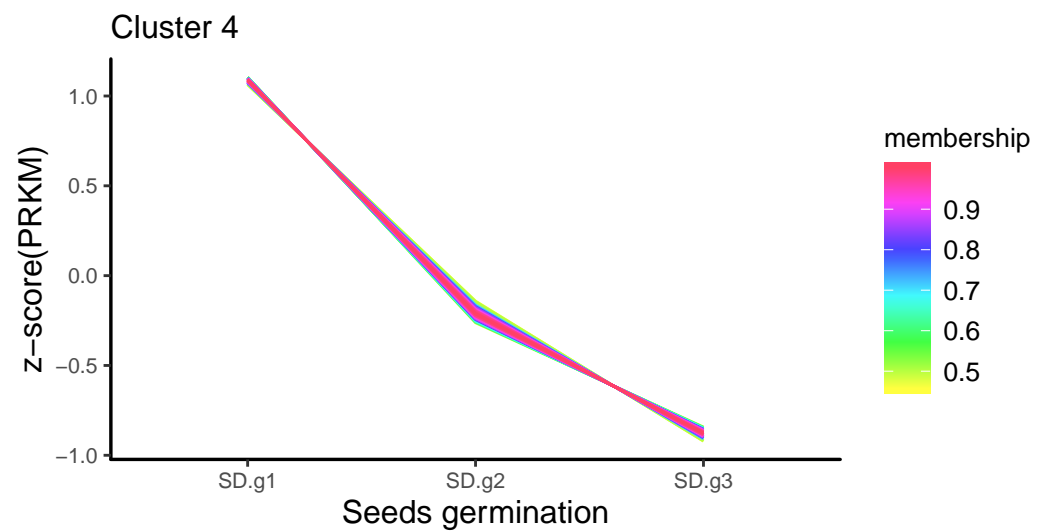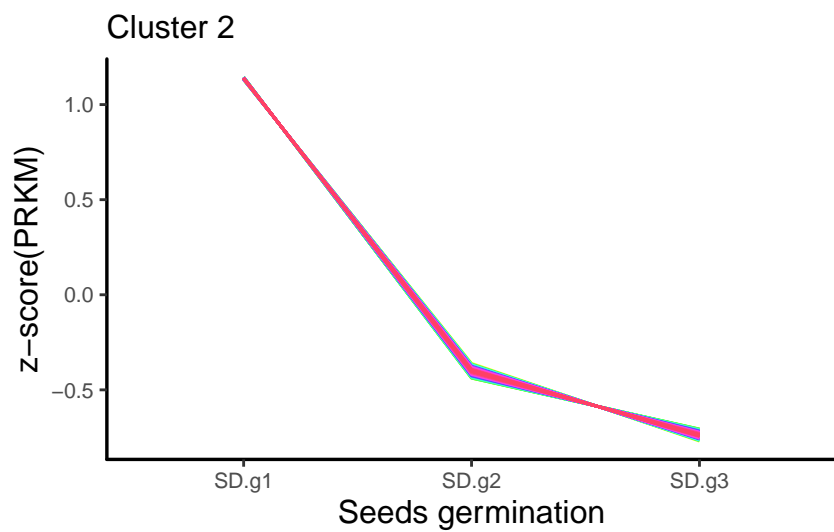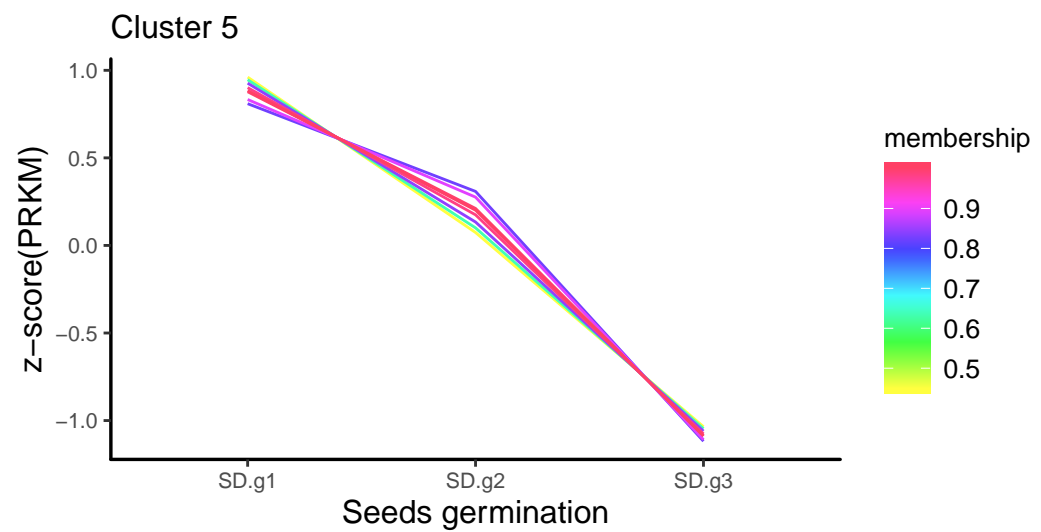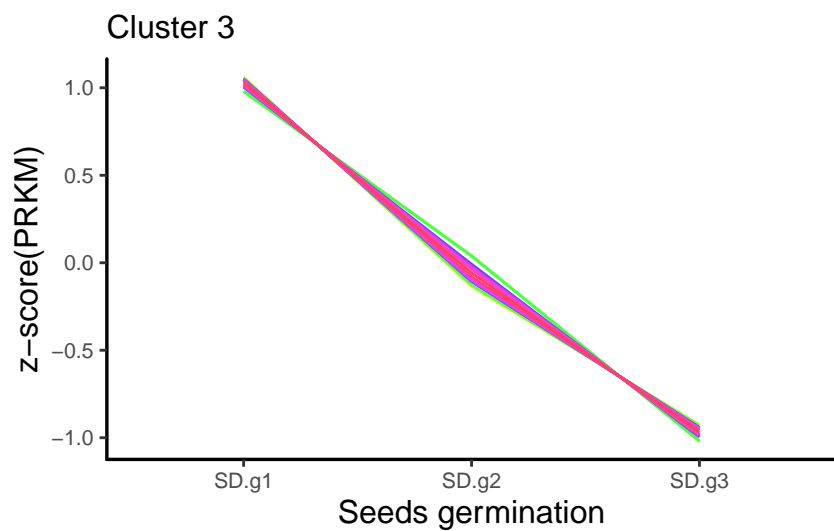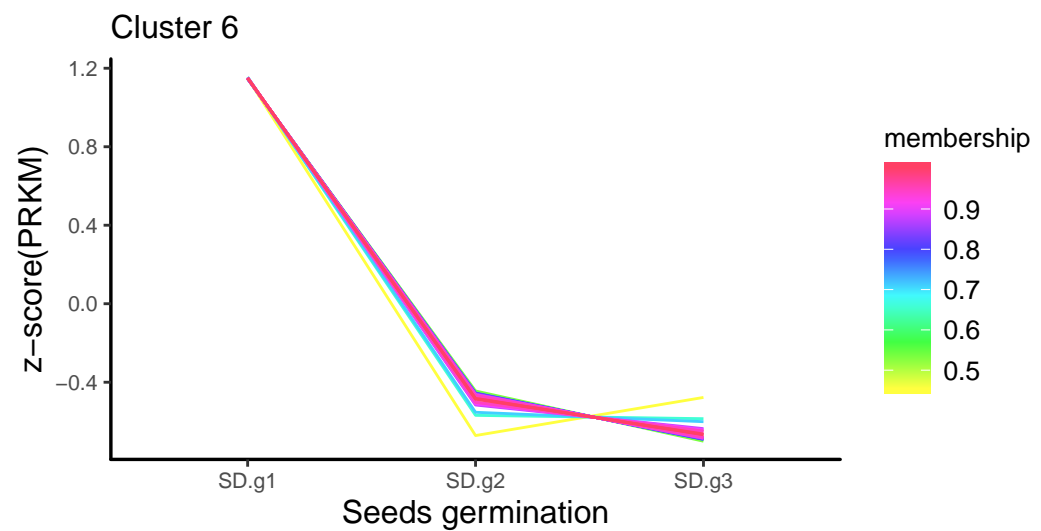

Supplement: Supplementary file 4 — Fig S4 [file PLD3-5-e00320-s010.pdf]

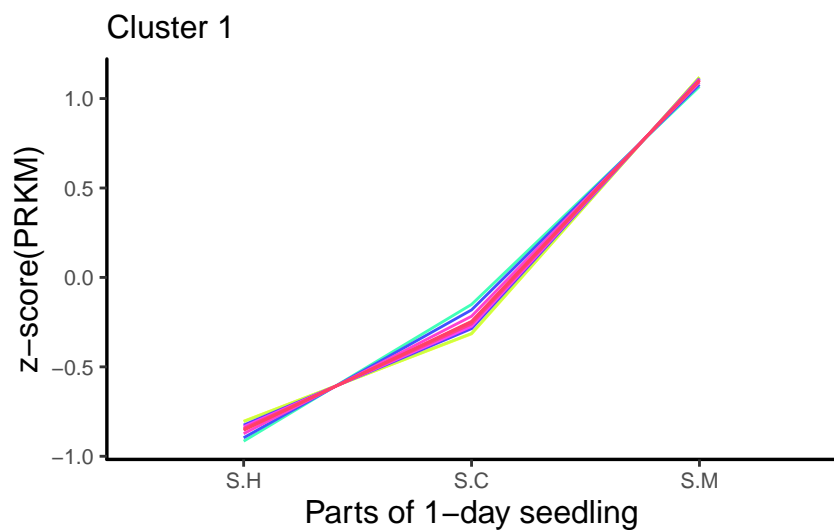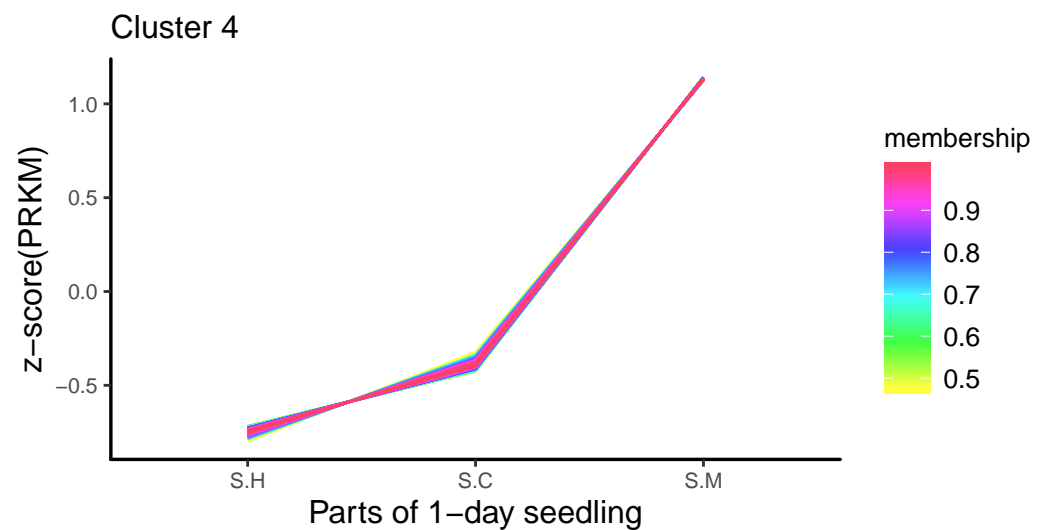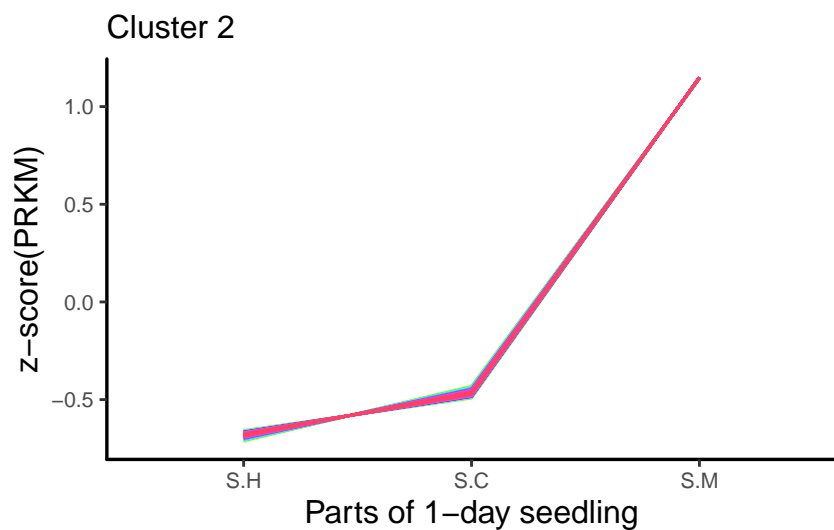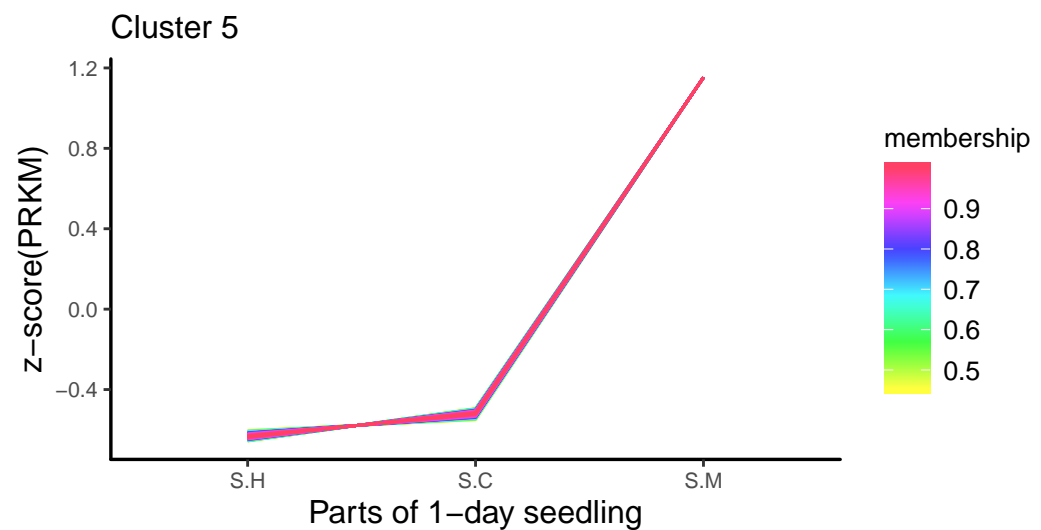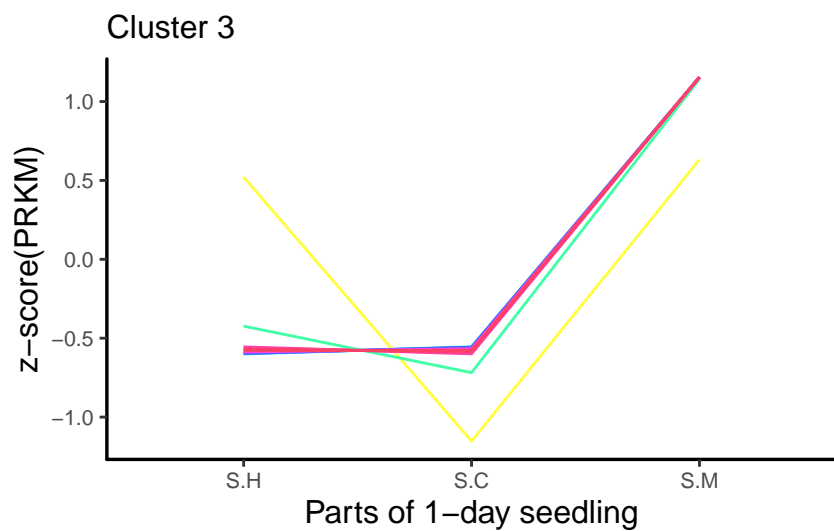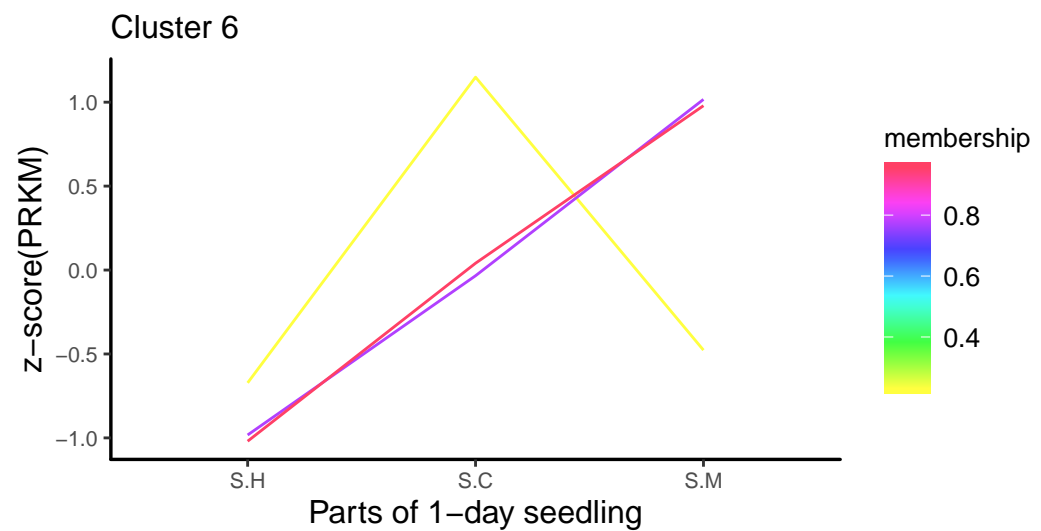

Supplement: Supplementary file 5 — Fig S5 [file PLD3-5-e00320-s004.pdf]
